# Exploring the Mystery of Angiotensin-Converting Enzyme II (ACE2) in the Battle against SARS-CoV-2

**DOI:** 10.1155/2021/9939929

**Published:** 2021-06-04

**Authors:** Divakar Sharma, Juhi Sharma, Amit Singh

**Affiliations:** ^1^Hericure Healthcare Pvt Ltd, Pune, India; ^2^Department of Microbiology, Maulana Azad Medical College, 110002, New Delhi, India; ^3^School of Basic and Applied Science, Eklavya University, M.P., 482002, Damoh, India; ^4^CCRF, All India Institute of Medical Sciences, New Delhi 110029, India

## Abstract

COVID-19 is the newly born pandemic caused by the SARS-CoV-2 virus, which is the recently emerged betacoronavirus that crosses the species barrier. It predominantly infects pneumocytes of the respiratory tract, but due to the presence of angiotensin-converting enzyme II (ACE2) on other cells like surface enterocytes of the upper esophagus and colon, these are also considered as the primary sites of infection. ACE2 receptor served as a cellular entry point for SARS-CoV-2. The expression of the ACE2 receptors is regulated by several factors such as age, tobacco smoking, inflammatory signaling, ACE inhibitors, angiotensin receptor blockers, and comorbidities (chronic obstructive pulmonary disease (COPD), tuberculosis, cerebrovascular disease, coronary heart disease, hypertension, and diabetes). Therefore, scientists are trying to explore the in-depth knowledge of ACE2 and considered it as a potential indirect target for COVID-19 therapeutics. In this focused review, we discussed in detail ACE2 expressions and regulation by different factors in the primary or vulnerable sites of SARS-CoV-2 infections. Clinical trials of rhACE2 in COVID-19 patients are ongoing, and if the outcome of the trials proves positive, it will be a breakthrough for the management of COVID-19. Finally, we suggest that targeting the ACE2 (a master regulator) in a balanced way could serve as a potential option against the management of COVID-19.

## 1. Introduction

Since the 21^st^ century, three species of genus *β*-coronaviruses (SARS-CoV, MERS-CoV, and SARS-CoV-2) have crossed the species barrier and caused atypical pneumonia (severe acute respiratory syndrome, Middle East respiratory syndrome, and COVID-19) in humans. The SARS and MERS outbreaks were epidemics, while recently, the emerged COVID-19 has become a pandemic. The SARS (2002) and MERS outbreaks (2012) first emerged in China and Saudi Arabia, respectively, which spread worldwide promptly and lead to hundreds of deaths with a fatality rate of 10% and 37%, respectively [1, 2]. Severe acute respiratory syndrome coronavirus-2 (SARS-CoV-2) has recently emerged in December 2019 at Wuhan province in China, spreading across the globe and caused COVID-19. The World Health Organization (WHO) declared COVID-19 as a pandemic on March 11, 2020, and worldwide, there were more than 140 million confirmed cases with more than 3 million deaths globally (April 21^st^, 2021) [3]. SARS-CoV-2 predominantly infects lower airways of the respiratory tract and causes mostly respiratory system-related issues, systemic illness, and bronchopneumonia-like conditions in 10-15% of patients [4]. However, less common extrapulmonary manifestations are also visible like diarrhea, headache, nausea, hemoptysis, and vomiting. Diarrhea manifestation was also detected in up to 30% and 10.6% of patients with MERS-CoV and SARS-CoV, respectively [5–7]. Among them, diarrhea is the early phase of manifestation in coronavirus infections, which is presumably due to viral replication in the gastrointestinal tract. In this focused review, we discussed ACE2 expressions in the primary or vulnerable sites of SARS-CoV-2 infections and regulation of ACE2 by a variety of factors such as age, tobacco smoking, inflammatory signaling, ACE inhibitors, angiotensin receptor blockers, and comorbidities.

## 2. Cellular Entry of SARS-CoV-2

SARS-CoV-2 (betacoronavirus) is a large size virus (120 nm in diameter) with positive-senses-RNA, encapsulated by the glycoprotein spikes, and causes severe infections in humans. Coronavirus spike (S) glycoprotein forms homotrimers, which interact with the host cell receptors (angiotensin-converting enzyme II/ACE2) and mediate cellular entry [8]. Host receptor-mediated cellular entry is not precise for all betacoronaviruses. MERS-CoV is the betacoronavirus that does not recognize the ACE2 receptor, while HCoV-NL63 (alphacoronavirus) recognizes the ACE2 receptor for cellular entry [9, 10]. SARS-CoV and SARS-CoV-2 both share the receptor-binding motifs (RBM) of the spike to interact with the ACE2 receptor of the host and could mediate the cellular entry [11, 12]. Coronavirus cellular entry is the series of events that requires the intensive action of receptor binding followed by proteolytic processing of the S protein to promote virus-cell fusion. S^B^ domain of SARS-Co-V recognized the ACE2 receptor of pneumocytes shares 75% amino acid sequence similarity with SARS-CoV-2 S^B^ domain and 50% similarity within the receptor-binding motifs (RBMs) [12, 13]. The S protein binding to ACE2 in SARS-CoV-2 is much stronger (10-20 folds superior) than the SARS-CoV, which reported an earlier epidemic in 2002-2003 [13, 14]. Researchers reported that the presence of furin cleavage sites at the S1/S2 boundary of the spike of SARS-CoV-2 is the novel attribute in contrast to SARS-CoV. Through *in vitro* experiments, they also reported abolition of the furin cleavage motif moderately affected SARS-CoV-2 S-mediated entry into VeroE6 or BHK cells [13]. A recent *in silico* study exposed that the SARS-CoV-2–ACE2 complex has shown a higher number of contacts, a larger interface area, and decreased interface residue fluctuations comparative to the ACE2 complex with other members of coronaviruses (SARS-CoV, HCoV-NL63) [15]. Furin and TMPRSS2 (transmembrane protease serine 2) are also other host cellular enzymes involved in the priming of coronavirus spike proteins [7, 11]. Recent research has shown that ACE2 is differentially regulated in certain circumstances (cigarette smoking) at RNA and protein levels and suggested that its RNA and protein levels are generally correlated [16].

## 3. Most Vulnerable Primary Sites for SARS-CoV-2 Infection

Theoretically, the presence of ACE2 receptors in numerous cells of the human body could be the potential sites of SARS-CoV-2 infection. ACE2 receptors are majorly expressed in epithelial alveolar lining of pneumocyte cells of the respiratory system as well as the upper esophagus, and colon (surface enterocytes of the small intestine) of the gastrointestinal system; therefore, these cellular sites are considered as the primary targets of SARS-CoV infection [11, 17, 18]. These receptors are also expressed in epithelial cells of the testis, liver, kidney, heart, blood vessels, etc. [19]. A study recently reported that the ACE2 expression in various organs and tissues at mRNA level might be the potential sites for coronaviruses infection [19]. Cardiovascular comorbidities also emerged rapidly as a major threat that potentiates COVID-19 because the cardiac pericytes expressed the higher ACE2 receptors which could be mediating the entry of the SARS-CoV-2 [19]. A recent study reported the presence of SARS-CoV-2 within endothelial cells and the gathering of inflammatory cells which subsequently lead to inflammatory cell death and suggests that viral infection facilitates the induction of endotheliitis in multiple organs as well as the host inflammatory response [20].

ACE2 receptors on the surface enterocytes of the small intestine primarily involved the absorption of the dietary amino acids and maintaining the homeostasis of the gut microbiota by regulating the expression of antimicrobial peptides [21]. It is hypothesized that SARS-CoV-2 infection is likely to cause an imbalance of permeability in the intestine, which results in enterocytemal-absorption. Studies suggested that ACE2 alteration and modification caused by SARS-CoV-2 could increase intestinal inflammation and diarrhea, which is similar to the earlier reports of SARS and MERS infections [21].

## 4. Factors Affecting the ACE2 Expression May Potentiate the Risk of COVID-19

SARS-CoV-2 can infect people of any age, but people with age over 55 years and with comorbidities like COPD, cerebrovascular disease, coronary heart disease, hypertension, tuberculosis, tobacco smoking, and diabetes are under a higher risk to develop COVID-19 [22–26]. A bunch of studies revealed that people with these comorbidities have shown the altered expression of the ACE2 receptors which might potentiate the SARS-CoV-2 infection and the severity of COVID-19 [27–29].

Several studies suggested that cigarette smoking is strongly associated with adverse outcomes from COVID-19 [30, 31]. An *in vivo* study on a mouse model suggested that the overexpression of ACE2 mRNA showed a shorter life span in SARS-CoV-infected mice [28]. These results provide the clue that increased expression of the ACE2 might increase the risk of SARS-CoV-2 infection. ACE2 receptors mediate the entry into the cell of three strains of coronaviruses (SARS-CoV, NL63, and SARS-CoV-2) [16]. ACE2 downregulation induced by viral invasion may be especially detrimental in people with baseline ACE2 deficiency associated. ACE2 downregulation induced by the cell entry of SARS-CoV, NL63, and SARS-CoV-2 may be particularly detrimental in subjects with preexisting ACE2 deficiency. Moreover, SARS-CoV-2 causes ACE/ACE2 balance disruption and RAAS activation, which leads ultimately to COVID-19 progression, especially in the patient with comorbidities, such as hypertension, diabetes mellitus, and cardiovascular disease [32, 33]. In human genetics, cohort studies give an insight into the relationship between RAAS and acute lung injury. It was found that polymorphisms in the ACE gene are associated with outcomes in ARDS [34]. Several epidemiological studies on smoking men and elderly patients have shown the severity of COVID-19 which could provide a clear-cut clue and correlation between the overexpression of ACE2 and severity of COVID-19 [30, 31]. A study of 1,099 COVID-19 patients admitted to an intensive care unit (ICU) showed that smokers (12.3%) needed ventilation as compared to nonsmokers (4.7%) [22].

Lung epithelial cells are the first line of defense against inhaled pathogens and are the target for many respiratory pathogens like viruses; thus, the dysregulation of endocytosis in these cells may affect viral susceptibility. A recent study has shown the overexpression of the gene of ACE2 in the epithelia of lower airways of smokers and COPD patients which might increase the probability of the SARS-CoV-2 infection [31]. Smokers and individuals with COPD have increased airway expression of ACE2, which is the entry receptor for SARS-CoV-2. Gene expression levels of ACE2 in the airways of individuals with and without COPD have been analyzed and found that COPD and current smokers had significantly increased expression of ACE2 [31, 35]. Therefore, upregulation of ACE2 may be useful in protecting the host against acute lung injury; chronically, this may predispose individuals to increased risk of coronavirus infections, which uses this receptor to gain entrance into epithelial cells. Active cigarette smoking and COPD upregulate ACE2 expression in lower airways, which may partially explain the increased risk of severe COVID-19 in these populations. Most recently, Smith et al. reported that in mammals, the lung ACE2 expression level is not affected by age or sex of the patients but smokers exhibited the overexpression of the ACE2 by the various lung cell types, including the secretory lineage or the secretory cells [36]. They have shown that chronic smoke exposure triggers the expansion of the secretory cell population and a concomitant increase in ACE2 expression in the respiratory tract. These researchers also demonstrate that inflammatory signaling triggered the ACE2 expression which can be upregulated by viral infections or interferon treatment [36]. Cytokine storms are the other major cause of COVID-19 showing the higher levels of circulating inflammatory cytokines. Therefore, these facts partially explain why smokers have a higher risk of SARS-CoV-2 infection; so we hypothesized that smoking might regulate the overexpression of ACE2 which could increase the risk of the COVID-19 as well as the severity of the diseases.

## 5. ACE2 May Be an Indirect Target for COVID-19 Therapeutics

The ACE2 acts as a master regulator of the renin-angiotensin system (RAS), which converts angiotensin II (Ang II) to Ang 1-7more kinetically than Ang I to Ang 1-9 [37]. Studies suggested the pathophysiological role of ACE2 in SARS, COVID-19, lung diseases, and other comorbidities [19, 24]. ACE2 has shown the protective effect in coronavirus-induced tissue injuries and could be considered as a potential indirect target for therapeutics [38]. An *in vivo* animal study has shown that a catalytically active form of ACE2 abrogates lung damage not only due to the direct action in the lungs but also through the ACE2-dependent gut-lung axis [39, 40]. COVID-19 patients may also have shown the symptoms of diarrhea earlier than the respiratory one. This gastrointestinal leakage and gut dysbiosis may have been linked to the onset of pH through the gut-lung axis and is closely related to hyperactivation of the ACE/angiotensin II type 1 receptor axis from ACE2 loss [40].

**Figure 1:**
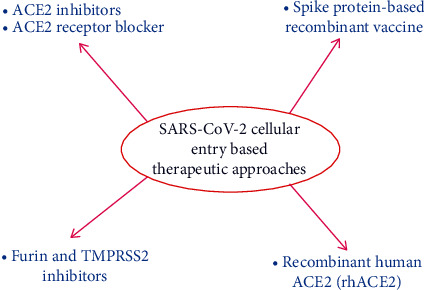
SARS-CoV-2 cellular entry based potential therapeutic approaches.

ACE inhibitors and angiotensin receptor blockers (ARBs) regulate the ACE2 expression in various tissues and organs through Ang II that could modulate the effects of RAS blockade on ACE2 [41, 42]. Recombinant human ACE2 (rhACE2; APN01, GSK2586881) is effective and safe in healthy volunteers and a small cohort of patients with ARDS and COVID-19 [41–43]. Its administration rapidly decreased Ang II and IL-6 concentration in plasma. Clinical trials of rhACE2 in COVID-19 patients are ongoing (Clinicaltrials.gov #NCT04287686). sACE2 is not only to influence viral invasion but also serves as a ligand to sequester SARS-CoV-2 away from mACE2 which internalizes docked viruses via membrane-associated enzyme dynamics that determines SARS-CoV-2 tissue tropism. Apart from this, sACE2 levels can cleave circulating angiotensin-II to Ang-(1-7) and increase the systemic protective effects of ACE2/Ang-(1-7)/Mas axis signaling to reduce disease morbidity. A probiotic species *Lactobacillus paracasei* can be engineered to express rhACE2 with the fusion of nontoxic subunit B of cholera toxin (used as a carrier to facilitate transmucosal transport) and used to treat mice with diabetic retinopathy [44]. This bioengineered probiotic species could potentially be repurposed to treat COVID-19 (Figure 1).

## 6. Conclusion and Future Perspective

SARS-CoV-2 is the causative agent of COVID-19, which predominantly infects pneumocytes of the respiratory tract. However, due to the presence of ACE2 on the other body cells like surface enterocytes of the upper esophagus and colon, these cells are also considered as the primary sites of infection. ACE2 receptor served as a cellular entry point for SARS-CoV-2. The expression of the ACE2 receptors is controlled by a variety of factors such as age, tobacco smoking, inflammatory signaling, ACE inhibitors, angiotensin receptor blockers, and comorbidities (COPD, tuberculosis, cerebrovascular disease, coronary heart disease, hypertension, and diabetes). Therefore, researchers are exploring the mystery of ACE2 and could consider it as a potential indirect target for COVID-19 therapeutics. Clinical trials of rhACE2 in COVID-19 patients are ongoing, and if the outcome of the trials proves positive, it will be used to manage COVID-19. Finally, we suggest that carefully targeting the ACE2 (master regulator) in a balanced way might be considered as a potent option against the management of COVID-19.
